# Complete Genomic Sequence of Dengue Virus Serotype 4 Isolated from Plasma Collected from a Haitian Child in 2014

**DOI:** 10.1128/genomeA.01160-17

**Published:** 2017-10-05

**Authors:** Maha A. Elbadry, Sarah K. White, Julia C. Loeb, Massimiliano S. Tagliamonte, Marco Salemi, Valery Madsen Beau De Rochars, Taina Telisma, Mohammed Rashid, J. Glenn Morris, John A. Lednicky

**Affiliations:** a Department of Environmental and Global Health, College of Public Health and Health Professions, University of Florida, Gainesville, Florida, USA; b Emerging Pathogens Institute, University of Florida, Gainesville, Florida, USA; c Department of Infectious Diseases and Pathology, College of Veterinary Medicine, University of Florida, Gainesville, Florida, USA; d Department of Pathology, Immunology and Laboratory Medicine, College of Medicine, University of Florida, Gainesville, Florida, USA; e Department of Health Services Research, Management and Policy, University of Florida, Gainesville, Florida, USA; f Christianville Foundation School Clinic, Gressier, Haiti; g Department of Medicine, College of Medicine, University of Florida, Gainesville, Florida, USA

## Abstract

While data are limited, there is increasing evidence that infections by dengue viruses are endemic in Haiti. In 2014, an outbreak caused by dengue virus 4 (DENV-4) followed a chikungunya fever outbreak. We present here the complete genome sequence of one isolate grouped within the genotype II South America and Caribbean DENV-4 clades.

## GENOME ANNOUNCEMENT

Dengue fever (DF) is the most prevalent arbovirus disease in the world (1, 2). There are five dengue virus (DENV) serotypes (3) that have been identified, four of which (4–6) are known to cause typical clinical manifestations associated with DF in humans. Strains of dengue virus serotypes 1 (DENV-1) to 4 (DENV-4) are known to circulate in the Caribbean, including Haiti (7, 8). However, little is known about their epidemiology or genomic diversity.

During a research investigation of the causes of acute undifferentiated febrile illness, we detected consecutive outbreaks caused first by DENV-1 and then by DENV-4, which followed in the aftermath of the major 2014 chikungunya fever epidemic (9). As previously reported, the studied population consisted of a cohort of children from a school clinic in the area of Gressier, Ouest Department, Haiti, and a diagnosis of DF was confirmed by virus isolation (3) and reverse transcription-PCR (3, 4).

For this report, the virus was isolated in cell line MRC-5 from one patient and was Sanger sequenced using a genome-walking approach to obtain a complete DENV-4 genomic sequence. Briefly, Accuscript high-fidelity reverse transcriptase (Agilent Technologies) and reverse primers were used to generate cDNAs, followed by PCR using Phusion Polymerase (New England Biolabs) and specific primers to produce overlapping amplicons for sequencing. The 5′ and 3′ terminals of the virus genome were amplified for sequencing using a rapid amplification of cDNA ends (RACE) kit (Life Technologies, Inc.).

As of August 2017, four genotypes (I to IV) of DENV-4 have been identified (4, 6). Genomic analyses of our isolate, designated DENV-4 strain Haiti/0324/2014, indicate that it groups with DENV-4 genotype II, which is a Caribbean and South American clade. The genomic sequence has 99.7% identity with another 2014 DENV-4 isolate from Haiti (GenBank accession number KP140942) and 97.4% identity with a 1994 isolate from Haiti (GenBank accession number JF262782).

### Accession number(s).

The complete genome sequence of DENV-4 strain Haiti/0324/2014 has been deposited in GenBank under accession number KT276273.
